# Potential effects of regular use of antihypertensive drugs for in-hospital delirium in geriatric patients with trauma

**DOI:** 10.1038/s41598-022-17182-3

**Published:** 2022-07-26

**Authors:** Hiroki Nagasawa, Kazuhiko Omori, Soichirou Ota, Ken-ichi Muramatsu, Kouhei Ishikawa, Youichi Yanagawa

**Affiliations:** Department of Acute Critical Care Medicine, Shizuoka Hospital, Juntendo University, 1129 Nagaoka, Izunokuni City, Shizuoka, 410-2295 Japan

**Keywords:** Outcomes research, Geriatrics

## Abstract

Although the regular administration of antihypertensive drugs is a risk factor for falls in older adults, whether their anti-inflammatory effects confer a survival benefit in older adults remains unknown. This single-center retrospective cohort study examined patients with trauma aged ≥ 65 admitted to our hospital between January 2018 and December 2020. Patients who had not received antihypertensive drugs before admission (i.e., AHT(−) group) and those who had received the drugs (i.e., AHT(+) group) were compared using a 1:1 propensity score-matched analysis. The primary outcome was 28-day mortality, and the secondary outcomes were in-hospital mortality and the incidence of complications during the hospital stay. In total, 637 patients were analyzed. After propensity score matching, each study group had 223 patients. No significant difference was observed in the primary outcome (28-day mortality: AHT(−) group, 3.6% vs. AHT(+) group, 3.6%; adjusted relative risk: 1.00, 95% confidence interval (CI): 0.38–2.62); only the in-hospital incidence of delirium was significantly low in the AHT(+) group (25.1% vs. 13.9%; adjusted relative risk: 0.55, 95% CI: 0.37–0.82). Overall, the regular use of antihypertensive drugs did not affect outcomes in geriatric trauma patients; however, the incidence of delirium was reduced in those regularly receiving antihypertensive drugs.

## Introduction

The world population is continuously aging, including in Japan. Older adults have high trauma mortality because of various factors such as multidrug administration, medical history, muscle weakness, poor nutrition, and weakened immunity^1–5^. Additionally, even if the trauma is mild, complications (e.g., infections, embolism, and delirium) might result in death or prolonged hospital stay^5–9^. Delirium is one of the unfavorable complications that bother not only patients themselves but also medical staff and patients’ families. A variety of factors can cause delirium, and it is mentioned that the systemic inflammatory response caused after trauma increases the risk of delirium, especially for older patients^9–11^.

On the one hand, the regular administration of antihypertensive drugs is a risk factor for falls in older adults^12–15^. Conversely, antihypertensive drugs are shown to exhibit anti-inflammatory effects^16^. Thus, it is not clear if the anti-inflammatory effects of antihypertensive drugs confer a survival benefit in older adults. Particularly in patients with sepsis, a better prognosis has been reported in those regularly taking antihypertensive drugs^17–20^. To the best of our knowledge, the relationship between the administration of oral antihypertensive drugs and mortality after hospitalization in patients with trauma has not yet been investigated. The first important point of our study is to clarify the potential effects of antihypertensive drugs used by many older patients. The second is to explore clues for preventing geriatric trauma with various complications.

In this study, we investigated the potential effects of antihypertensive drugs in patients with trauma by evaluating the 28-day mortality and the incidence of complications and hypothesized that those receiving antihypertensive drugs might have a better prognosis than those not receiving these drugs.

## Methods

### Study design and setting

Our institution delivers critical trauma care to an area with a population of 1,500,000 people. This observational single-center retrospective cohort study was conducted in accordance with the Declaration of Helsinki, and it followed the Strengthening the Reporting of Observational Studies in Epidemiology (STROBE) guidelines^21^.

### Ethical considerations

The protocol for this study was approved by the Medical Ethics Committee of the Juntendo University Shizuoka Hospital (approval number E21-0089). The requirement for obtaining patient consent was waived owing to the study’s retrospective nature.

### Selection of participants

We collected the data of older (≥ 65 years) patients with trauma admitted to our hospital between January 2018 and December 2020. Patients admitted for cardiac arrest, burns, toxins, asphyxiation, near-drowning, or hanging were excluded. Moreover, patients who transferred to other hospitals within 3 days of admission and those with unclear medical and drug histories were excluded.

### Data collection

Collectors who were not among the authors used a medical chart review to collect data, including patient demographics, medical history, injury severity score (ISS), vital signs upon arrival (i.e., Glasgow coma scale score, systolic blood pressure, heart rate, respiratory rate, and body temperature), complications during hospital stay (i.e., acute kidney injury [AKI], acute respiratory distress syndrome [ARDS], arrhythmia, bleeding, cardiovascular disease, delirium, infections, stroke, and venous thromboembolism [VTE]), “do not attempt resuscitation (DNAR)” order, invasive treatments received within 24 h of admission (i.e., blood transfusion, emergency operation under general anesthesia, interventional radiology, tracheal intubation), and 28-day in-hospital mortality. The white blood cell (WBC) count and C-reactive protein (CRP) values collected as inflammation values were the highest three days after injury. Supervisors checked and anonymized the data collected. The authors subsequently analyzed the data.

### Definition

We defined “antihypertensive medication” as a prescription of antihypertensive drugs and their administration within 28 days before the injury. Antihypertensive drugs were defined as angiotensin-converting enzyme (ACE) inhibitors, angiotensin II receptor blockers (ARBs), β blockers, calcium channel blockers (CCBs), and thiazides. We ascertained antihypertensive information from pharmacy notebooks or medical information documents from patients’ family doctors.

### Outcomes

The primary outcome was 28-day mortality, which was selected with reference to the results of previous studies^17–20^. The secondary outcomes were in-hospital mortality and the incidence of complications (i.e., AKI, ARDS, arrhythmia, bleeding, cardiovascular disease, delirium, infections, stroke, and VTE) during the hospital stay. These variables were selected based on their impact on the prognosis of trauma patients after admission.

### Statistical analysis

AHT(-) group comprised individuals who did not take any antihypertensive drug (as per their medical records) for > 28 days before admission and those who were not prescribed antihypertensive drugs. AHT(+) group comprised those who had clear information of antihypertensive drugs and had not recorded information of drug withdrawal before admission. These AHT(−) and AHT(+) groups were compared using a 1:1 propensity score-matched analysis. Logistic regression analysis was used to estimate the propensity scores. The variables included in the model were age, sex, type of injury, vital signs on arrival, ISS, DNAR order, treatments received within 24 h upon admission, and comorbidities. We used a caliper with a width of 0.2 of the standard deviation of the logit of the propensity score. The balance between both groups was evaluated using the standardized mean difference (SMD), with an SMD > 0.1 indicating a significant imbalance. Data are presented as the median and interquartile range or numbers and percentage, as appropriate. Thereafter, outcomes were compared in the matched cohort. The relative risk (RR) was calculated, and the differences and 95% confidence intervals (CIs) were reported.

In the sub-analysis, to assess the effects of each antihypertensive drug on delirium, multiple-logistic regression analysis was performed in the cohort group. Odds ratio (OR) was calculated, and the differences and 95% CIs were reported. A univariate analysis was performed to investigate the relationship between delirium and the number of antihypertensive drugs.

As a post-hoc analysis to evaluate the association between delirium and inflammation, we collected inflammation data, and the Kruskal–Wallis and Steel–Dwass tests were performed in four groups according to the presence or absence of delirium and antihypertensive medication. The four groups were as follows: A, AHT(+) and delirium(+); B, AHT(−) and delirium(+); C, AHT(+) and delirium(−); D, AHT(−) and delirium(−).

We used Fisher’s exact test, Mann–Whitney U test, and logistic regression analysis. Missing values were handled using pairwise methods. All statistical analyses were performed using EZR 1.54 (Saitama Medical Center, Jichi Medical University, Saitama, Japan), a graphical user interface for R 4.0.2 (The R Foundation for Statistical Computing, Vienna, Austria)^22^. For the sensitivity analysis, the original cohort (n = 637) as a first sensitivity analysis and an ISS ≥ 16 cohort as a second sensitivity analysis that was also performed were matched and evaluated with the same outcomes. Statistical significance was set at *P* < 0.05 or based on the 95% CI.

## Results

### Patient characteristics

**Figure 1:**
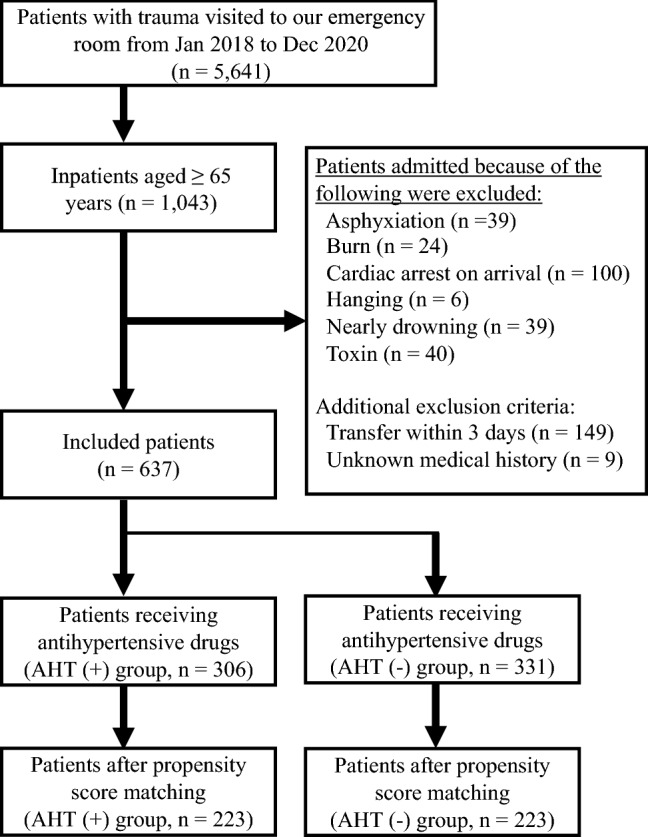
Flow chart of participant inclusion.

In total, 5,641 patients with trauma were treated in our emergency room, of which 1,043 satisfied the inclusion criteria. After excluding 406 patients, 637 were analyzed in our study. Based on the medication history, we divided this population into two groups: the AHT(−) and AHT(+) groups. In this cohort, the AHT(+) group included duplication of antihypertensive drugs, 17 ACE inhibitors (5.6%), 175 ARBs (57.2%), 65 β blockers (21.2%), 203 CCBs (66.3%), and 28 thiazides (9.2%). Using the propensity score estimated by a multivariable logistic regression analysis of 637 patients, we obtained 223 in each group (Fig. 1). The c-statistic for the goodness-of-fit model was 0.70 (95% CI: 0.66–0.74). After the propensity score matching, the AHT(+) group included duplication, 11 ACE inhibitors (4.9%), 132 ARBs (59.6%), 43 β blockers (19.3%), 148 CCBs (66.3%), and 16 thiazides (7.2%). Table 1 shows the baseline characteristics of the patients before propensity score matching. In the matched population, there were no significant differences in the baseline characteristics of patients between the two groups (Table 2).

**Table 1:** Baseline characteristics of older trauma patients with or without antihypertensive drugs.

Characteristics	AHT(−) group (n = 331)	AHT(+) group (n = 306)	*P*-value	SMD
Age, year	75 [70, 82]	78 [72, 84]	< 0.001	0.273
**Sex, *n* (%)**			0.570	0.050
Male	205 (61.9)	182 (59.5)		
Female	126 (38.1)	124 (41.5)		
**Type of injury, *n* (%)**			0.302	0.092
Blunt	321 (97.0)	301 (98.4)		
Fall	201 (60.7)	214 (69.9)		
Traffic accident	105 (31.7)	81 (26.5)		
Others*	15 (4.5)	6 (2.0)		
Penetrating	10 (3.0)	5 (1.6)		
Injury severity score	10 [5, 17]	9 [5, 16]	0.156	0.091
**Vital signs upon arrival**				
Systolic blood pressure, mmHg	144 [120, 162]	148 [128, 164]	0.143	0.116
Heart rate, bpm	80 [69, 93]	81 [70, 93]	0.633	0.007
Respiratory rate, bpm	20 [17, 23]	20 [16, 21]	0.125	0.160
Glasgow coma scale	15 [14, 15]	15 [14, 15]	0.137	0.039
Body temperature, °C	36.5 [36.2, 36.8]	36.5 [36.1, 36.8]	0.223	0.003
**Comorbidities, *n* (%)**				
Autoimmune disease	8 (2.4)	8 (2.6)	1.000	0.013
Chronic heart failure	7 (2.1)	13 (4.2)	0.172	0.122
Chronic kidney disease	10 (3.0)	30 (9.8)	< 0.001	0.280
Chronic liver disease	8 (2.4)	11 (3.6)	0.486	0.069
Chronic lung disease	14 (4.2)	18 (5.9)	0.368	0.075
Dementia	22 (6.6)	22 (7.2)	0.876	0.021
Diabetes mellitus	50 (15.1)	80 (26.1)	0.001	0.275
Malignancy	17 (5.1)	16 (5.2)	1.000	0.004
Old myocardial infarction	8 (2.4)	28 (9.2)	< 0.001	0.291
Psychiatric disease	10 (3.0)	10 (3.3)	1.000	0.014
Stroke	20 (6.0)	52 (17.0)	< 0.001	0.348
DNAR order, *n* (%)	2 (0.6)	4 (1.3)	0.435	0.072
**Treatment received within 24 h upon admission, *n* (%)**				
Blood transfusion	69 (20.8)	60 (19.6)	0.767	0.031
Emergency operation	49 (14.8)	50 (16.3)	0.662	0.042
Interventional radiology	14 (4.2)	15 (4.9)	0.708	0.032
Tracheal intubation	42 (12.7)	34 (11.1)	0.625	0.049

Categorical variables are presented as counts and percentages (%), whereas numerical variables are presented as median and interquartile ranges. The balance between the two groups was evaluated using the SMD; an SMD > 0.1 was considered significant. *Others included crush, sports, animal-related, and abuse. *AHT* Antihypertensive drug, *DNAR* Do not attempt resuscitation, *SMD* Standardised mean difference.

**Table 2:** Characteristics of the study population after propensity score matching.

Characteristics	AHT(−) group (n = 223)	AHT(+) group (n = 223)	*P*-value	SMD
Age, year	77 [71, 83]	77 [72, 84]	0.723	0.037
**Sex, *n* (%)**			1.000	0.009
Male	130 (58.3)	129 (57.8)		
Female	93 (41.7)	94 (42.2)		
**Type of injury, *n* (%)**			1.000	0.036
Blunt	220 (98.7)	219 (98.2)		
Fall	136 (61.0)	157 (70.4)		
Traffic accident	73 (32.7)	57 (25.6)		
Others*	11 (4.9)	5 (2.2)		
Penetrating	3 (1.3)	4 (1.8)		
Injury severity score	9 [5, 17]	9 [4, 14]	0.426	0.057
**Vital signs upon arrival**				
Systolic blood pressure, mmHg	147 [124, 165]	148 [128, 164]	0.956	0.028
Heart rate, bpm	80 [68, 90]	81 [69, 91]	0.429	0.058
Respiratory rate, bpm	20 [16, 22]	20 [16, 22]	0.785	0.052
Glasgow coma scale	15 [14, 15]	15 [14, 15]	0.109	0.020
Body temperature, °C	36.5 [36.3, 36.8]	36.5 [36.1, 36.8]	0.268	0.090
**Comorbidities, *n* (%)**				
Autoimmune disease	5 (2.2)	4 (1.8)	1.000	0.032
Chronic heart failure	6 (2.7)	7 (3.1)	1.000	0.027
Chronic kidney disease	10 (4.5)	13 (5.8)	0.669	0.061
Chronic liver disease	7 (3.1)	7 (3.1)	1.000	< 0.001
Chronic lung disease	11 (4.9)	11 (4.9)	1.000	< 0.001
Dementia	16 (7.2)	18 (8.1)	0.859	0.034
Diabetes mellitus	45 (20.2)	41 (18.4)	0.719	0.045
Malignancy	13 (5.8)	13 (5.8)	1.000	< 0.001
Old myocardial infarction	8 (3.6)	8 (3.6)	1.000	< 0.001
Psychiatric disease	7 (3.1)	8 (3.6)	1.000	0.025
Stroke	20 (9.0)	21 (9.4)	1.000	0.016
DNAR order, *n* (%)	2 (0.9)	3 (1.3)	1.000	0.043
**Treatment received within 24 h upon admission, *n* (%)**				
Blood transfusion	69 (20.8)	60 (19.6)	0.767	0.031
Emergency operation	49 (14.8)	50 (16.3)	0.662	0.042
Interventional radiology	14 (4.2)	15 (4.9)	0.708	0.032
Tracheal intubation	42 (12.7)	34 (11.1)	0.625	0.049

Categorical variables are presented as counts and percentages (%), whereas numerical variables are presented as median and interquartile ranges. The balance between the two groups was evaluated using the SMD; an SMD > 0.1 was considered significant. *Others included crush, sports, animal-related, and abuse. *AHT* Antihypertensive drug, *DNAR* Do not attempt resuscitation, *SMD* Standardised mean difference.

### Main findings

**Figure 2:**
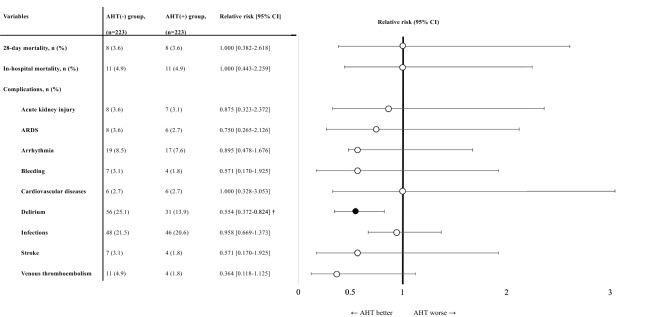
Primary and secondary outcome events. Relative risks of each outcome. Horizontal bars show 95% CIs. A thick vertical line represents a relative risk of 1.0, indicating no significant difference in outcome between AHT(−) and AHT(+) groups. † The results were statistically significant based on 95% CI, and a black circle plotted on the horizontal bar. Causes of death in the ATH(-) group were as follows: severe brain injury (n = 6), heart failure (n = 1), sepsis (n = 3), natural death (n = 1). Causes of death in the ATH(+) group were as follows: severe brain injury (n = 3), haemorrhage shock (n = 1), aortic dissection (n = 1), heart failure (n = 1), multiple organ failure (n = 1), sepsis (n = 2), malignant tumor (n = 2). AHT, antihypertensive drug; ARDS, acute respiratory distress syndrome; CI, confidence interval.

Figure 2 shows the outcomes of the matched cohort. No significant difference was observed in the primary outcome (i.e., 28-day mortality) between the two groups (AHT(−) group, 3.6% vs. AHT(+) group, 3.6%; adjusted RR: 1.00, 95% CI: 0.38–2.62). Regarding the secondary endpoints, only the in-hospital incidence of delirium showed a significant difference; it was significantly lower in the AHT(+) group than that in the AHT(−) group (25.1% vs. 13.9%; adjusted RR: 0.55, 95% CI: 0.37–0.82).

In the first sensitivity analysis performed in the original cohort (n = 627), there was no significant difference in 28-day mortality between the two groups (3.6% vs. 4.2%; RR: 1.17, 95% CI: 0.54–2.53). Like the main analysis, the incidence of delirium was also lower in the AHT(+) group (24.1% vs. 14.3%; RR: 0.64, 95% CI: 0.45–0.91) than in the AHT(−) group. In the second sensitivity analysis, 187 patients with an ISS ≥ 16 were identified from the study population, and 55 were matched in both groups. Some variables (age, sex, comorbidities of dementia, and diabetes mellitus) were not balanced between the two groups even after matching. Like the main analysis, there was no significant difference in 28-day mortality between the two groups (10.9% vs. 10.9%; adjusted RR: 1.00, 95% CI: 0.34–2.9). The incidence of delirium was also lower in the AHT(+) group (46.9% vs. 19.1%; adjusted RR: 0.39, 95% CI: 0.20–0.77) than in the AHT(−) group (Table 3).

**Table 3:** Outcome events of sensitivity analyses.

Variables	RR [95% CI] in original cohort (n = 637)	RR [95% CI] in ISS ≥ 16 cohort (n = 110)
28-day mortality	1.172 [0.543–2.529]	1.000 [0.344–2.910]
In-hospital mortality	1.226 [0.623–2.412]	1.000 [0.404–2.474]
**Complications**		
Acute kidney injury	1.475 [0.688–3.161]	1.000 [0.263–3.798]
ARDS	0.721 [0.299–1.740]	1.000 [0.307–3.261]
Arrhythmia	1.172 [0.688–1.996]	1.714 [0.730–4.027]
Bleeding	0.927 [0.315–2.728]	0.750 [0.176–3.196]
Cardiovascular diseases	1.487 [0.606–3.649]	1.333 [0.313–5.682]
Delirium	0.640 [0.452–0.907] †	0.391 [0.199–0.768] †
Infections	0.884 [0.648–1.205]	1.100 [0.683–1.771]
Stroke	0.787 [0.321–1.930]	4.000 [0.462–34.657]
Venous thromboembolism	0.721 [0.299–1.740]	1.000 [0.146–6.848]

The RRs to each variable for the use of antihypertensive drugs were evaluated. † The results were considered statistically significant based on 95% CI. *ARDS* Acute respiratory distress syndrome, *ISS* Injury severity score, *CI* Confidence interval, *RR* Relative risk.

### Sub-analysis

In the sub-analysis, ORs of each antihypertensive drug for the incidence of delirium were as follows: ACE inhibitors, OR: 0.83, 95% CI: 0.22–3.13; ARBs, OR: 0.58, 95% CI: 0.33–1.01; β blockers, OR: 1.26, 95% CI: 0.62–2.56; CCBs, OR: 0.76, 95% CI: 0.47–1.24; and Thiazides, OR: 1.35, 95% CI: 0.48–3.80 (Table 4). RRs of the antihypertensive drugs for the incidence of delirium were as follows; none (RR: 1.33, 95% CI: 1.14–1.57), one (RR: 0.65, 95% CI: 0.41–1.04), and more than one (RR: 0.80, 95% CI: 0.55–1.16).

**Table 4:** Sub-analysis; ORs of each antihypertensive drugs for delirium.

Variables	OR [95% CI]
ACE inhibitors	0.833 [0.222–3.130]
ARBs	0.581 [0.333–1.010]
β blockers	1.260 [0.621–2.560]
CCBs	0.763 [0.469–1.240]
Thiazaides	1.350 [0.481–3.800]

The ORs of each kinds of antihypertensive drugs for incidence of delirium were evaluated. † The results were considered statistically significant based on 95% CI. *ACE* Angiotensin-converting enzyme, *ARB* Angiotensin II receptor blocker, *CCB* Calcium channel blocker, *CI* Confidence interval, *OR* Odds ratio.

### Post-hoc analysis

**Figure 3:**
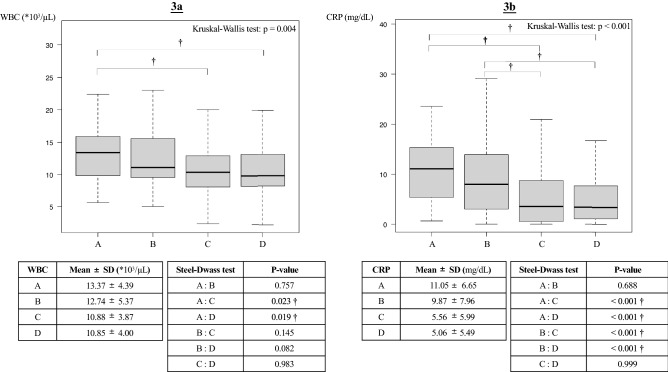
Result of post-hoc analysis. The four groups are as follows: A, AHT(+) and delirium(+); B, AHT(−) and delirium(+); C, AHT(+) and delirium(−); D, AHT(−) and delirium(−). The WBC count and CRP values were the highest in three days after injury. The vertical dashed lines between the horizontal bars show 95%CIs. The black thick horizontal bars are the mean value, and gray boxes are SDs. † The results were statistically significant based *p*-value < 0.05. We performed the Kruskal–Wallis test, a non-parametric analysis, because the homoscedasticity of the data rejected. (**a**) Significant differences were observed between groups A and C and groups A and D (Kruskal–Wallis test: *p* = 0.004). (**b**) Significant differences were observed between groups A and C, A and D, B and C, and B and D (Kruskal–Wallis test: *p* < 0.001). AHT, antihypertensive drug; CI, confidence interval; CRP, C-reactive protein; SD, standard deviation; WBC, white blood cell.

Both WBC and CRP values showed an almost similar trend (Fig. 3). Although there was no statistically significant difference in the value of WBC count and CRP with or without antihypertensive drugs, the value of inflammation in the first three days after admission trend to be higher in groups A and B with delirium.

## Discussion

To our knowledge, the relationship between the use of antihypertensive drugs and mortality or morbidity rates after an injury has not yet been investigated. Thus, this is the first study to show that antihypertensive drug usage may not affect mortality in older patients with trauma, although it may reduce the risk of delirium during hospitalization.

Previous studies on sepsis showed that using antihypertensive drugs reduced the risk of in-hospital mortality^17–20^. However, our results were inconsistent. One possible reason for this is the small number of outcomes; it might have reduced the effect size and curbed the required difference. The in-hospital mortality rate in our study population was approximately 5%; however, previous studies reported 30- to 90-day mortality rates of approximately 20–40%. In retrospect, a study of patients with a cluster of severe traumas (ISS ≥ 16) may be helpful, because the mortality of each group was 10.9% in this cohort. The difference in mortality among patients with an ISS ≥ 16 was also examined, despite the insufficient detection ability of the results owing to the small study population size. Alternatively, the degree of systemic inflammation may have been small, and the anti-inflammatory effect of antihypertensive drugs may not have affected the mortality of the patients with trauma.

Delirium is a clinically influential event that commonly occurs in hospitalized geriatric patients. No evidence is available to support the direct effects of antihypertensive drugs on the suppression of delirium risk because there has been no previous study on the direct relationship between the use of antihypertensive drugs and delirium^23^. Harrison et al. reported the difference in the rate of delirium depending on the type of antihypertensive drug. However, the comparison was not examined with patients who did not take antihypertensive drugs^23^. Zaal et al. investigated the risk factors for developing delirium in patients in the intensive care unit, and hypertension was mentioned as one of the factors. However, it was unclear whether patients had been taking antihypertensive drugs^24^.

One possibility of reducing the incidence of delirium is the anti-inflammatory effect of antihypertensive drugs^16^. Moreover, a systemic inflammatory response is induced after trauma^25^. Conversely, systemic inflammation and endothelial dysfunction are involved in delirium^26–28^. In our post-hoc analysis, the group with antihypertensive drugs did not suppress the increased inflammatory response after injury. Furthermore, the inflammatory response trend was significantly high in the group complicated with delirium. The fact that the values of the inflammatory response caused by trauma were the same between the AHT(+) and AHT(−) groups means that the protective effects of antihypertensive drugs on endothelial function have been speculated to suppress endothelial dysfunction, resulting in a reduced risk of delirium. One basis for this hypothesis is the incidence of VTE, which has also been reported to result from an acute inflammatory response and endothelial damage after trauma^29^. Although we did not find any statistically significant difference, the risk of VTE tended to be lower in the AHT(+) group. In this study, it was not possible to show the difference in the effects of delirium depending on the type or number of antihypertensive drugs administered. Studies on larger cohorts would be needed to better understand this.

Consequent complications, such as delirium^30,31^, worsen the prognosis of geriatric patients with trauma^8^. Therefore, the prevention of delirium is important. Although the use of antihypertensive drugs before injury may have a preventive effect on delirium, whether the re-administration or the initiation of antihypertensive drugs after hospitalization exhibits a preventive effect on delirium remains unknown. In polytrauma cases, older patients may likely experience hemorrhagic shock during the acute phase or may experience sudden exacerbation because of infection or re-bleeding, even after they have overcome the acute phase. Furthermore, the use of as-needed antihypertensive medication is associated with increased mortality and prolonged hospital stay^32^. Thus, there is no clear recommendation regarding resuming the oral administration of antihypertensive drugs. Further studies should investigate the appropriate time to resume the use of antihypertensive drugs and the mechanism underlying the preventive effect on delirium.

This study had some limitations. First, this was a retrospective cohort study conducted at a single center. Thus, we could not eliminate bias in patient backgrounds. Additionally, it is difficult to show a causal relationship in a retrospective study owing to the difficulty in isolating confounders; therefore, only the statistical relationship of association could be stated. Second, we used propensity score matching, which could not evaluate the effect of unknown variables that might have led to biased results. Third, data on patient compliance until the day before the injury are unavailable due to the retrospective study design. If patients have histories of oral antihypertensive drug administration without actually taking them, the interpretation of the results may be altered. Fourth, we could not cover all types of antihypertensive drugs. We narrowed down the section to five types, ACE-inhibitors, ARBs, CCBs, β blockers, and Thiazides, because we referred to previous studies^17–20^. There are many types of drugs classified as antihypertensive drugs, and there are other types besides the five antihypertensive drugs, such as α-blockers. Fifth, we could not investigate the cause of hypertension since it was difficult to obtain information due to the study’s retrospective design. Lastly, various antihypertensive drugs have been insufficiently investigated, and it is unclear which class of drugs had affected the results. Our cohort was too small to investigate the effects of various antihypertensive drugs individually.

In conclusion, the regular use of antihypertensive drugs did not affect outcomes in geriatric patients with trauma. However, the incidence of delirium might be reduced in patients with regular use of antihypertensive drugs. Our findings contribute to a growing body of evidence for improved care after admission for this vulnerable population and may provide new insights into medication management following admission.

## Data Availability

The dataset of our research has been given at https://doi.org/10.17632/yt5sf9yzf7.1.
